# Improvement of Supercapacitor Performance of In Situ Doped Laser-Induced Multilayer Graphene via NiO

**DOI:** 10.3390/nano13142081

**Published:** 2023-07-16

**Authors:** Nagih M. Shaalan, Shalendra Kumar, Faheem Ahmed, Nishat Arshi, Saurabh Dalela, Keun Hwa Chae

**Affiliations:** 1 Department of Physics, College of Science, King Faisal University, P.O. Box 400, Al-Ahsa 31982, Saudi Arabia; sjagdish@kfu.edu.sa (S.K.); fahmed@kfu.edu.sa (F.A.); 2 Physics Department, Faculty of Science, Assiut University, Assiut 71516, Egypt; 3 Department of Physics, School of Engineering, University of Petroleum & Energy Studies, Dehradun 248007, India; 4 Department of Basic Sciences, Preparatory Year Deanship, King Faisal University, P.O. Box 400, Al-Ahsa, 31982, Saudi Arabia; nishat.arshi8@gmail.com; 5 Department of Pure & Applied Physics, University of Kota, Kota 324005, India; sdphysics@rediffmail.com; 6 Beamline Research Division, Pohang Accelerator Laboratory, Pohang University of Science and Technology, Pohang 37673, Republic of Korea; khchae@kist.re.kr; 7 Advanced Analysis & Data Center, Korea Institute of Science and Technology, Seoul 02792, Republic of Korea

**Keywords:** NiO-doped graphene, laser-induced graphene, supercapacitor, electrochemical properties

## Abstract

Herein, we have reported a novel strategy for improving the electrochemical performance of laser-induced graphene (LIG) supercapacitors (SCs). The LIG was prepared using a CO_2_ laser system. The polyimide polymer was the source material for the fabrication of the LIG. The doping process was performed in situ using the CO_2_ laser, which works as a rapid thermal treatment to combine graphene and NiO particles. NiO was used to improve the capacitance of graphene by combining an electric double-layer capacitor (EDLC) with the pseudo-capacitance effect. The high-resolution transmission electron microscopy, energy-dispersive X-ray spectroscopy, and Raman spectroscopy showed that the structure of the LIG is multilayered and waved. The HRTEM image proves the distribution of NiO fine particles with sizes of 5–10 nm into the graphene layers. The electrochemical performance of the as-prepared LIG was tested. The effect of the combination of the two materials (oxide and carbon) was investigated at different concentrations. The LIG showed a specific capacitance of 69 Fg^−1^, which increased up to 174 Fg^−1^ for the NiO-doped LIG. The stability investigations showed that the electrodes were very stable for more than 1000 cycles. This current study establishes an innovative method to improve the electrochemical properties of LIG.

## 1. Introduction

It has been suggested that SCs are an alternative solution for single and hybrid applications compared with other storage devices [1]. In addition to being the best power source for electric and hybrid vehicles, SCs have many advantages including high energy density, long life, fast charge–discharge time, low input impedance, and environmental friendliness [2]. Several hybridization topologies have been proposed and applied over the past decade to increase the energy density and lifecycle of energy storage systems. The latest technology aims to put SCs in direct competition with rechargeable batteries [3]. SCs can store electrical energy as a solid tool to overcome several shortcomings of batteries. Thus, SCs have a promising future as they may become an alternative to energy storage batteries in many applications.

SCs are categorized into electric double layer capacitors (EDLC) [4] and pseudo-capacitors [5]. The capacitance of the EDLC results from the thin film accumulation of charges at the electrode–electrolyte contact area. For pseudo-capacitance, the energy is stored based on the Faradaic interactions between the electrolyte and the electrode, where the energy is stored in the form of chemical bonds [6]. Reversible and reducible interactions are responsible for these capacitors. Oxide materials have Faraday reactivity due to their low electrical conductivity. The irreversible faradaic reactions result in disappointing electrochemical performance and lower cyclic lifetimes. However, several supercapacitors may combine the two phenomena, allowing them to have a significant increase in capacitance and better stability than those with pseudo-capacitance.

Graphene material has interesting properties due to its high electrical conductivity, high surface area, mechanical strength, and lightweight [7,8]. Therefore, there have been many studies on the performance of graphene in the field of supercapacitors [9,10]. Pure graphene usually performs poorly as a micro-capacitor, but studies have revealed that atomically modified graphene can exhibit extraordinary performance. Graphene is composed of carbon atoms with sp^2^ hybrid orbitals in a hexagonal honeycomb lattice. The combination of these unique physicochemical properties makes graphene-based materials promising candidates for electrochemical energy storage devices [11,12,13,14]. The interface chemical reactions between the carbon materials and foreign elements modify its electronic structure. However, improving the electrochemical properties of graphene remains a challenge for researchers.

A method to produce multilayer graphene (laser-induced graphene (LIG)) from polyimide polymer films using CO_2_ lasers has been reported [15,16]. Laser technology has the advantages of being non-contact, non-toxic, rapid, and highly controllable technology. The surface area of LIG is 340–350 m^2^/g [15,16]. In addition to this advantage, LIG is fabricated using air from a commercial polymer sheet. Thus, laser technology provides a rapid path for fabricating energy storage devices. 

However, one must keep in mind that increasing the capacitance value does not mean only a modification of the intrinsic capacitance of the electric double layer. The higher capacitance of the capacitors may result from combining the pseudo-capacitance and EDLC mechanisms of charge storage. This type of capacitor is often confused with typical redox-based storage. In this work, we present an easy and simple method to improve the storage properties of graphene by merging NiO directly using CO_2_ laser technology. The structure of the LIG was studied using electron microscopy, EDX, and Raman spectrometry. LIG and doped LIG were investigated as supercapacitor electrodes. The performance of the LIG electrodes was significantly improved by NiO doping. An extensive study was carried to analyze the electrochemical properties of the manufactured electrodes. 

## 2. Materials and Methods

**Figure 1:**
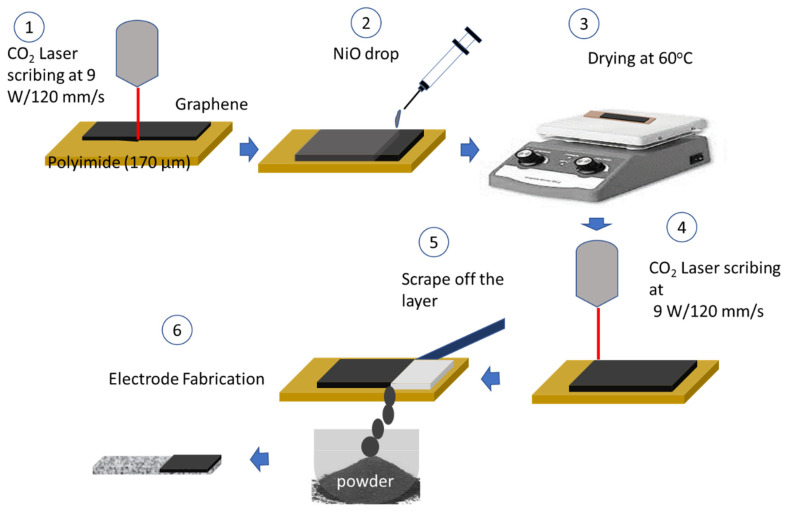
The illustration demonstrates the steps for preparing LIG and NiO-doped LIG.

The suspended NiO was prepared based on the prescribed NiO amount using ethanol. NiO nanopowder with a particle size of less than 50 nm was purchased from Sigma Aldrich and was used without further modification. An amount of 10 mg NiO was added to 5 mL of ethanol at a concentration of 2 mg/mL. The suspended solution of NiO was sonicated for 20 min before withdrawing the required amount. The laser system contained 40 W of the maximum power with a head speed of 400 mm/s. Polyimide sheets (TapeCase, Elk Grove Village, IL, USA) with a thickness of 5.0 mil (170 µm and 10 mm × 50 mm) were used as raw materials for LIG. To fabricate the LIG, the laser power was 9.0 W and the head speed was 120 mm/s. Then 1.0 mg/cm^2^, corresponding to 2.5 mL of NiO-suspended solution, and 2.0 mg/cm^2^, corresponding to 5.0 mL of NiO-suspended solution, were wisely dropped on 5.0 cm^2^ of the fabricated LIG sheet during drying at 60 °C on a hot plate. Afterward, the sheet was again subjected to the laser beam under the same conditions. The steps of the LIG preparation are shown in Figure 1. Finally, the LIG powders were collected from the surface of the polyimide. o prepare the electrochemical electrodes, a slurry was prepared by taking 80% LIG with 10% polyvinylidene difluoride (PVDF) as a binder and 10% conductive carbon black. All samples were mixed in an agate mortar with 1-methyl-2-pyrrolidone (NMP) solvent drops. Finally, a thick layer was coated on the foam, which was dried overnight at 80 °C. Three electrodes were prepared using the same steps. Based on pristine LIG and NiO-doped LIG with 1.0 mg/cm^2^ and 2.0 mg/cm^2^, three electrodes termed LIG-0, LIG-1, and LIG-2 were prepared, where LIG-0 stands for pristine LIG, LIG-1 stands for NiO-doped LIG with 1.0 mg/cm^2^ of NiO, and LIG-2 stands for NiO-doped LIG with 2.0 mg/cm^2^ of NiO.

A high-resolution transmission electron microscope (HRTEM: JEOL, JEM-2100F, Tokyo, Japan) working at 200 kV was used to analyze the products. For TEM measurements, the sample was dropped on a carbon-coated copper grid after the sonication in ethanol. Selected area electron diffraction (SAED) was carried out for the pristine and doped graphene. The Raman shift of the graphene was carried out using LabRAM-HR800 of a He-Cd laser with a wavelength of 633 nm. The supercapacitor measurements were measured using the CorrTest electrochemical workstation. The experiments were carried out in a three-electrode electrochemical cell including a counter electrode of Pt wire, a reference electrode of saturated calomel electrode (Ag/AgCl), and a working active electrode. The aqueous electrolyte was 1.0 M KOH. Within a frequency range of 0.1 Hz to 1.0 MHz, the electrochemical impedance spectrum (EIS) was examined. Various swipe voltages of 5 to 100 mVs^−1^ were used for measuring the cyclic voltammetry curves. The electrodes were charged and discharged (GCD) at current densities of 1.6 to 3.3 Ag^−1^.

## 3. Results and Discussions

### 3.1. Structure Analysis

**Figure 2:**
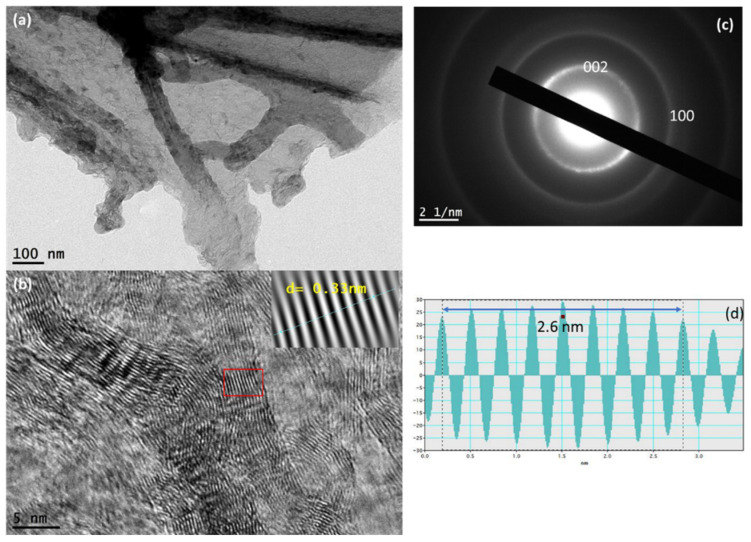
HRTEM analysis for LIG: (**a**) TEM image of a multilayer LIG; (**b**) HRTEM imaging of LIG lattice; (**c**) SAED for LIG; and (**d**) the intensity profiles of 8 atomic planes of LIG lattice.

**Figure 3:**
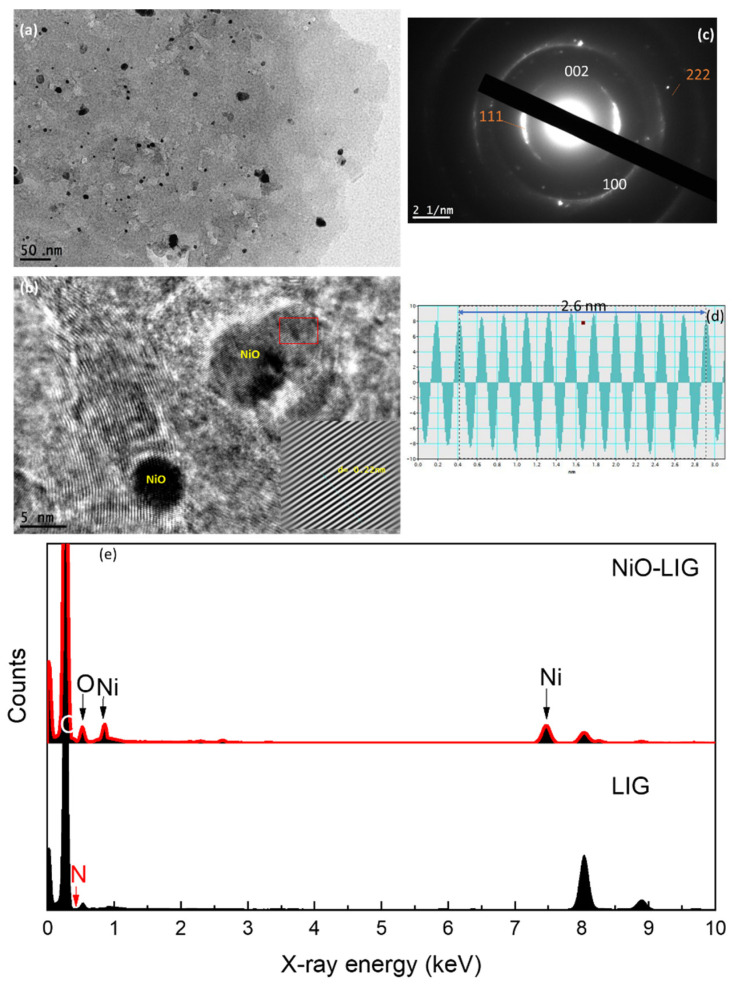
HRTEM analysis for NiO-doped graphene: (**a**) TEM image of NiO and multilayer LIG; (**b**) HRTEM imaging of LIG and NiO particles; (**c**) SAED for LIG and NiO particles; (**d**) the intensity profiles of 11 atomic planes of NiO lattice; and (**e**) EDX spectra for LIG and NiO-doped LIG.

**Figure 4:**
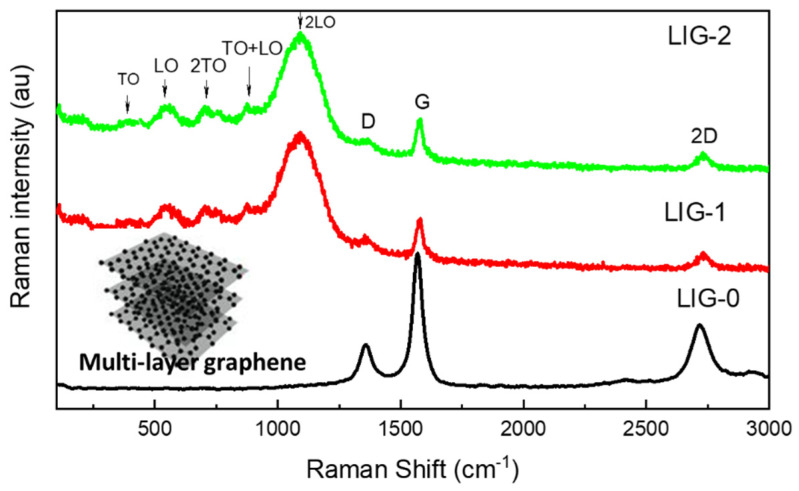
Raman spectra of LIG-0, LIG-1, and LIG-2, NiO, and multilayered graphene scattering modes.

Figure 2, Figure 3 and Figure 4 show a deep investigation of LIG using HRTEM and Raman spectrometry. The TEM image confirmed the multilayer phase of graphene fabricated by the CO_2_ laser. The lattice image of the LIG with a wide scale shown in Figure 2b exhibited that the LIG has curved ribbons or a curved structure with a width of 4–10 nm and lengthy ribbons. Also, this image confirmed the crystalline phase of LIG. The spacing between the atomic columns is also clear in the magnified inset image of Figure 2b. The LIG lattice planes show a d-spacing of 0.33 nm corresponding to the (002) plane structure [15]. However, the SAED pattern found in Figure 2c exhibited two different planes corresponding to (002) and (100) [15,17]. The intensity profile of the atomic planes showed a spacing of 2.6, corresponding to 8 atomic lines, confirming that the d-spacing is 0.33 nm for the selected lattice fringes. Also, the level of waving LIG of the atomic columns may be indicated from the vertices of the atomic planes in Figure 2d.

For more deep information about the NiO particle size and distribution on the graphene surface, HRTEM images were examined for the NiO-doped graphene sample, as shown in Figure 3. The TEM image shown in Figure 3a demonstrates the existence of individual and accumulated NiO particles in the graphene layers. The size of the NiO fine crystals was ~5–10 nm, as shown in the lattice image of Figure 3b, although some nanoparticles were aggregated to form large particles, as shown in Figure 3a. The d-spacing of the NiO particles is 0.23 nm, which corresponds to the crystal plane of NiO (111) [18,19]. Figure 3b proves that NiO fine particles are well attached to the surface of graphene. In the NiO-doped graphene sample, the SAED shows a mixed hollows of graphene and NiO. Compared to the diffraction planes of graphene, there are overlapping rings of NiO with diffraction spots, as shown in Figure 3c. Figure 3d exhibits the intensity profile of the NiO atomic planes; the spacing between 11 atomic planes showed that 2.6 nm corresponding to a d-spacing is 0.23 nm for the plane (111) of the selected lattice fringes. Figure 3e shows the EDX spectra of the LIG and NiO-doped LIG. In the LIG sample, only carbon atoms and partially oxygen atoms were detected. Although polyimide contains two acyl groups (C=O) bonded to nitrogen (N), N atoms were not detected in EDX, where N should be detected at an X-ray energy of 0.392 keV. For NiO-doped graphene, Ni atoms were detected at different X-ray energies, and an increase in oxygen intensity was observed.

The multilayer LIG was well defined by the Raman spectrum, where only three D-, G-, and 2D-bands were observed. These three bands are shown in the Raman spectrum im Figure 4. The graphitic structure and its level are defined by the G-band intensity, which exhibits a vibration mode that possesses E_2g_ of the sp^2^ bond in the Brillouin zone center [20]. The disorders or defects in the LIG lattice were expressed by the intensity of the D-band at 1358 cm^−1^, which could be assigned to disordered graphitic carbon and the presence of sp^3^ defects [18]. The second scattering of the D-band is defined by the 2D band and refers to two phonon scattering processes. The D-band, G-band, and 2D-band were observed at 1358, 1568, and 2721 cm^−1^ for the LIG. The Raman spectra of the NiO-doped LIG are also shown in Figure 4, where the NiO Raman spectrum showed broadening peaks at 380, 530, 730, 900, and 1097 cm^−1^ [21,22], while the LIG Raman spectra showed the same three peaks. The Raman spectra of LIG-1 and LIG-2 were almost similar. Since the Raman spectra are a surface-sensitive phenomenon, the NiO peaks dominate the Raman intensity. The dominance of NiO in the graphene Raman spectrum has also been reported [18]. Raman spectra are also sensitive to surface crystallinity. Thus, the broadening peaks of NiO prove that the NiO particles are very fine. The Raman scattering of NiO originates from one-phonon (transverse optical (TO) at 380–400 cm^−1^ and longitudinal optical (LO) at 530 cm^−1^) and two phonons (2TO at 730 cm^−1^, TO+LO at 900 cm^−1^, and 2LO at 1097 cm^−1^) excitations. For pristine LIG, the intensity ratios of the D/G and 2D/G bands are important parameters, which give more details about the nature of LIG, where I_D/G_ indicates the level of the defect in LIG, and I_2D/G_ indicates the number of layers. The I_D/G_ value was 0.29, which confirms the low lattice defects of the LIG prepared here [23,24,25]. The multilayer graphene was confirmed where I_2D/G_ showed a value of 0.43, which is less than 1.0 [25,26].

### 3.2. Electrochemical Characterizations

**Figure 5:**
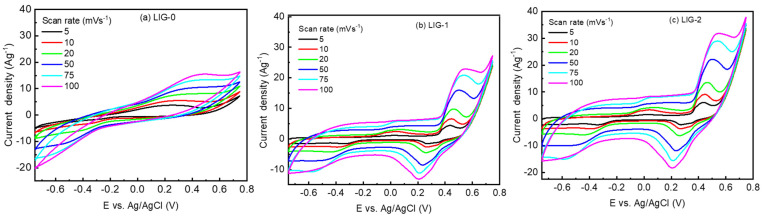
CV curves of LIG-0, LIG-1, and LIG-2 electrodes at scan rates of 5 up to 100 mVs^−1^.

Electrochemical measurements were carried out in a three-electrode configuration in an alkaline KOH electrolyte. The application was carried out for the three prepared electrodes (LIG-0, LIG-1, and LIG-2) under the same conditions to evaluate their performance. Cyclic voltammetry (CV) curves were investigated at various scan rates between 5 and 100 mVs^−1^, as shown in Figure 5. The suitable potential window was −0.75 to 0.75 V. In the case of LIG-0, the CV was a square-like curve, and no redox peak was observed, as shown in Figure 5a. For the electrodes doped with 1.0 mg/cm^2^ of NiO, redox peaks appeared at a value of 0.52 and 0.2 V. There was also a broadening in the region between 0.4 and −0.7 V, as shown in Figure 5b. This indicates an intermediate effect on the electrode due to NiO fine particles. By increasing the amount of the NiO-suspended solution, the CV curves of the LIG-2 electrodes showed a broadening redox peak in the increasing direction and decreasing direction at the same position as LIG-1. The maximum peaks can be defined at 0.45 V and shifted to 0.55 V by increasing the scan rate from 5 to 100 mV/s. The reduction peaks were also observed at 0.3 V and shifted to 0.2 V, as shown in Figure 5c. It is clear from these changes that NiO doping using the current method has a synergetic effect on the electrochemical behavior of LIG. The area under the CV curves in VA increased, indicating an increase in the specific capacitance.

**Figure 6:**
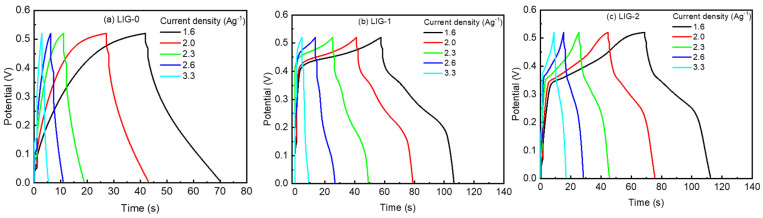
GCD curves for (**a**) LIG-0, (**b**) LIG-1, and (**c**) LIG-2 at various current densities.

The behavior observed in the above CV curves was reflected and shown in the galvanostatic charge–discharge (GCD) curves, as shown in Figure 6. The GCD curves of LIG-0 behave exactly like carbon materials, where the symmetry in the charging and the discharge curves was observed, as shown in Figure 6a. The result confirmed the EDLC storage mechanism of the LIG-0 electrode. The GCD was completely different in the case of the LIG-1 and LIG-2 electrodes, as shown in Figure 6b. This difference was more pronounced in the case of the LIG-2 electrode, as shown in Figure 6c. The behavior observed in these two electrodes may combine the pseudo-capacitance and EDLC mechanisms, where LIG and NiO are combined in the same sample. In general, all capacitors were charged with different current densities of 1.6 to 3.3 Ag^−1^, where the higher current density exhibited faster charging, and vice versa. The maximum charging–discharging time of LIG-0 at 1.6 Ag^−1^ was 70 s, while it was 107 and 112 s for LIG-1 and LIG-2, respectively. However, it was observed that the LIG-2 electrode took a longer time (67 s) to be charged compared to LIG-1 (57 s), which took a longer time compared to the LIG-0 electrode (41 s). The observed behavior was due to the chemical modification of the LIG by NiO, where physical adsorption between the LIG material and NiO nanoparticles occurred. Thus, the electrode has a performance of oxidation and carbon materials, where the oxide and carbon materials perform both the pseudo-capacitance and EDLC mechanisms. The scribing of NiO by the laser generated a modification for the surface of the LIG. Thus, an increase in the charge capacity of the modified LIG by 200–300% compared to that of the raw LIG was observed.

**Figure 7:**
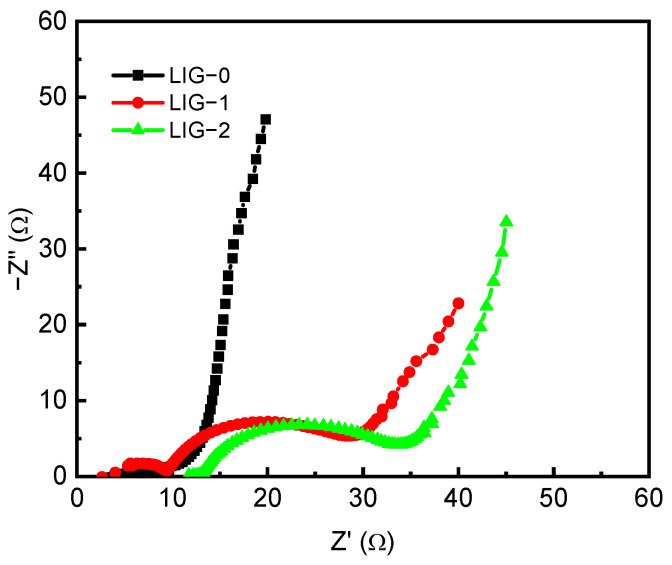
Nyquist plots of LIG-0, LIG-1, and LIG-2.

The electrical impedance spectroscopy (EIS) was measured in the range of 0.1 Hz to 1.0 MHz. The three electrodes presented typical AC impedance attributes of supercapacitors. Figure 7 shows the EIS curves of LIG-0, LIG-1, and LIG-2 electrodes. The high-frequency region represents two messages: (1) the x-intersection represents the common resistance (R_s_) containing the resistance of the electrode materials, the ionic resistance of the electrolyte, and the contact resistance between the electrode and the current collector; and (2) the radius of the semicircle represents the electrode conductivity and the charge transfer resistance (R_ct_) of the electrode material. In the middle-frequency region, the oblique curve represents the Warburg impedance, which represents the diffusion/transport of ions in the electrolyte. The capacitive behavior of the electrode takes place on the curve in the low-frequency region. As we mention later, the electrochemical properties of graphene changed significantly due to NiO doping, as the capacitance increased to 174 F/g. The modeled values of R_s_ and R_ct_ withdrawn from the Nyquist diagram are listed in Table 1. The Rs resistance increased for the electrodes doped by NiO, where it was 3 Ω for LIG-0 and increased to 10 and 13 Ω for LIG-1 and LIG-2, respectively. The Rc_t_ also showed a large change after doping, where it increased from 5 up to 22 Ω. The interesting is the semi-circle, which was largely demonstrated at a higher frequency for the doped electrodes. It was expected that in the case of pure LIG, the electrode would work as an EDLC supercapacitor, as indicated in the CV and GCD curves. In the EDLC mechanism, the electrolyte ions accumulate on the electrode surface to form a double layer charge. However, in the LIG doped with NiO, both pseudo-capacitance and EDLC mechanisms occurred.

**Figure 8:**
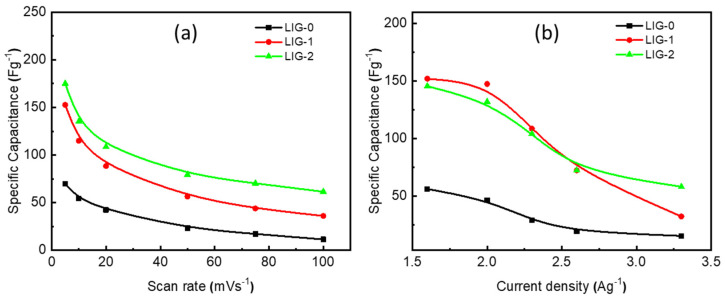
The calculated specific capacitance from (**a**) CV curves and (**b**) GCD curves for LIG-0, LIG-1, and LIG-2 electrodes.

The specific capacitance of graphene has been previously reported to be 75, 99, and 135 Fg^−1^ when ionic liquid, organic, and aqueous electrolytes were used [27,28]. Figure 8 exhibited the specific capacitance as a function of the swipe voltage for LIG-0, LIG-1, and LIG-2. LIG-0 demonstrated a low specific capacitance compared to LIG-1 and LIG-2. The specific capacitance was 69 Fg^−1^ at 5 mVs^−1^ for LIG-0 and decreased with an increase in the scan rate. The specific capacitances of the LIG-1 and LIG-2 were 152 Fg^−1^ and 174 Fg^−1^, respectively. It was observed that the NiO-doped LIG was still higher than that of the pristine LIG at all scan rates. Figure 8b shows the specific capacitance calculated from the GCD curves. It was observed that the specific capacitance was independent of the NiO concentration. However, a threefold improvement in the specific capacitance was recorded when the LIG was doped by NiO. The specific capacitances at a discharge current density of 1.6 Ag^−1^ are 150, 148, and 52 Fg^−1^ for LIG-2, LIG-1, and LIG-0, respectively. With increasing the discharging current up to 3.3 Ag^−1^, the specific capacitances were 58, 32, and 15 Fg^−1^ for LIG-2, LIG-1, and LIG-0, respectively.

**Figure 9:**
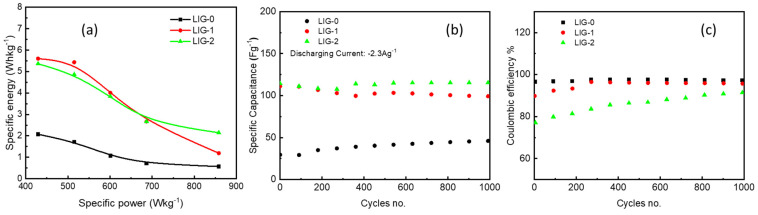
(**a**) Specific energy versus specific power, (**b**) specific capacitance versus cycles, and (**c**) Coulombic efficiency (CE) versus cycles for LIG-0, LIG-1, and LIG-2 electrodes.

Supercapacitors, electrochemical capacitors, double layer capacitors, or Farad capacitors are electrochemical components for storing energy through a symmetric electrode. It is a kind between a conventional capacitor and a battery, adopting a double layer and a pseudo-capacitor to store electrical energy. It has a high electrical power compared to batteries, which have a high energy. Often, in capacitors, there is no chemical reaction during the energy storage process, and thus it is reversible and enables us to recharge and discharge thousands of times. Supercapacitors have fast charging speeds and can reach more than 95% of their capacity within a short time. Figure 9 presents some of the most important properties inherent in the process of the electrochemical properties of supercapacitors, such as the relationship between energy and electrical power, cyclic stability, and coulombic efficiency. The specific energy as a function of the specific power of the electrodes is recorded in Figure 9a. At a current density of 1.6 Ag^−1^ and a specific power of 429 Wkg^−1^, the specific energy has maximum values of 5.3, 5.6, and 2.0 Whkg^−1^ for LIG-2, LIG-1, and LIG-0, respectively. The specific energy of the NiO-doped LIG increased about three times compared to that of LIG-0. With increasing current density, the specific energy decreased, while the specific power increased.

The specific capacitance measured for 1000 cycles of charging and discharging at 2.3 Ag^−1^ is shown in Figure 9b. The cyclic stability test is an important parameter in supercapacitors to demonstrate the stability of electrodes [29]. The electrodes showed good stability during the repeatability of charge–discharge cycles. It is observed that the specific capacitance value of the LIG-0 increased in the first 200 cycles from the value of 29 and stabilized at 37 Fg^−1^ for the rest of the cycles. As shown in Figure 9b, the capacitance of the LIG-2 electrode remained stable over 1000 cycles without change, the electrode did not deteriorate, and still showed high stability compared to the LIG-1 electrode, which displayed a very slight decrease. In general, the stability of the electrodes in the electrochemical processes performed in an aqueous electrolyte is dependent on various factors, including corrosion of the surface of the electrode, infiltration of the electrode material into the body of the electrolyte, and some secondary chemical reactions that occur between the electrolyte and the electrode [29]. In addition to the irreversible Faradaic interactions that also contribute to the deterioration or decomposition of the electrodes, which may be achieved in the present case in the LIG-1 and LIG-2 electrodes, the pseudo-capacitance process is combined. Faradaic reactions may contribute at least 10% of the decomposition of the electrolyte [30]. The increase in LIG-0 capacitance could indicate two reasons: (1) it may result from a slight oxidation process that occurs on the surface of graphene during the charging and discharging processes; and (2) it may be due to the hydrophobic properties of LIG. Thus, the aqueous electrolyte slowly penetrates the graphene layers and reaches LIG. When the penetration is completed, stability occurs, as shown in Figure 9b. An important factor in supercapacitors is the Coulombic efficiency (CE) of the electrodes in the GCD curves. The CE is defined as the ratio between charging and discharging charges (Qdischarging/Qcharging), which is the same ratio between the time of discharging and time of charging at a constant current density. The CE values for different cycles is shown in Figure 9c at a current density of 2.3 Ag^−1^. It is obvious that LIG-0 started with high efficiency at 95% and continued at almost the same CE until the 1000th cycle. For LIG-1, the CE started at 90% for LIG-1 and improved with the 300th cycle to 96% to the 1000th cycle. For LIG-2, the CE value was 77% and gradually improved to 92% at the 1000th cycle. Despite doping graphene with a material that exhibits a Faradaic reaction, the electrodes still show a good electrochemical performance in terms of cycling, charging, and discharging.

The doping mechanism of NiO-doped graphene through the CO_2_ laser can be proposed. As shown in the CVs of LIG-0, LIG-1, and LIG-2 presented in Figure 5, only the CVs of NiO-doped graphene have two redox reaction peaks that come from the redox of NiO (NiO and NiOOH) in the KOH electrolyte solution $\left(NiO+OH^{-}↔NiOOH+e^{-}\right)$ [31,32]. Thus, extra charges could be stored by the oxide through electrochemical redox reactions. The LIG-1 and LIG-2 electrodes have larger VA (voltammetric current) responses compared to LIG-0, exposing higher energy storage. Due to the behavior of the pseudocapacitive occurring in the reversible Faradaic reactions of Ni(II)/Ni(III), the capacitance was enhanced. Also, the redox peaks become slightly higher for LIG-2 compared to LIG-0, which may indicate that the electrode performance was slightly improved by increasing the Ni^2+^ concentration due to the increase in NiO/graphene, which exposes the pseudocapacitive behavior. However, it is expected that there will be a trade-off between the conductivity (based on LIG, which is a highly conductive support to the electrode) and pseudocapacitance (based on NiO) in the presence of extra NiO, which may lead to an increase in the resistance and reduce the current. It is observed in Figure 6 that the increase in NiO causes an increase in charging time compared to the discharge time for NiO-doped graphene, reducing the Coulombic efficiency. Also, the capacitive performance was almost similar for both NiO concentrations.

Table 2 shows the previously reported results for supercapacitors with LIG electrodes. The areal capacitances and the specific capacitances were reported based on pristine LIG or the doped LIG electrodes. In Table 2, the supercapacitor parameters such as capacitance, power density, and energy of similar electrodes are presented. The areal capacitance of the LIG and its doped phase ranged between 29 and 995 mF/cm^2^ at different charging currents or scan rates of the swipe voltage. Also, the high specific capacitance of LIG-PEDOT recorded a value of 115 F/g, compared to the present value of 174 F/g for NiO-doped LIG.

## 4. Conclusions

In summary, we summarize an easy strategy to improve the capacitive performance of graphene by incorporating nickel oxide into its layers using direct laser technology. NiO fine particles of less than 50 nm were dropped on the fabricated graphene and scribed using a CO_2_ laser. HRTEM confirmed the fabrication of multilayered graphene sheets using a CO_2_ laser. It has been clearly shown that the crystalline planes appear (002) mostly in this type of graphene, with a d-space of 0.33 nm. Also, through SAED, it was found that this graphene contained two planes of (002) and (100). The fabrication of multilayer graphene and NiO-doped graphene sheets was also confirmed by Raman spectra, where D, G, and 2D of graphene and scattering peaks of NiO. Electrodes with different concentrations of NiO were tested for their electrochemical properties as supercapacitors without any additional processing. A significant improvement was observed in the performance of the composite electrodes as the specific capacitance increased from 69 to 174 Fg^−1^ at a scanning rate of 5 mVs^−1^. The same performance was also observed in the case of the specific capacitance calculated from the charge and discharge curves, which had that the two concentrations of NiO showed almost the same specific capacitance values. In all electrodes, the lifecycle performance was excellent up to the thousandth cycle. The stored energy increased 3 times (from 2.0 to 5.6 Whkg^−1^ corresponding to a power of 429 WKg^−1^). We can conclude that the current combination strategy between oxide and graphene is suitable for improving the electrochemical properties of graphene and can be used for further studies in the future.

## Figures and Tables

**Table 1:** The values R_s_ and R_ct_ for the investigated electrodes.

Sample	Rs (Ω)	Rct (Ω)
LIG-0	3	5
LIG-1	10	18
LIG-2	13	22

**Table 2:** Comparison between supercapacitor electrodes fabricated by LIG a with polyimide sheet.

Active Material	Capacitance	Energy	Power	Ref.
LIG	800 µF/cm^2^ at 10 mV/s	--	--	[33]
LIG	34 mF/cm^2^ at 0.1 mA/cm^2^	1.0 mWh/cm^3^	11 mW/cm^3^	[34]
LIG	995 mF/cm^2^ at 1 mA/cm^2^	55.9 µWh/cm^2^	9.39 mW/cm^2^	[35]
LIG+MoS_2_+MnS	58.3 mF/m^2^ at 50 mA/cm^2^	7 µWh/cm^2^	49.9 µW/cm^2^	[36]
NiO/Co_3_O_4/_LIG	29.5 mF/cm^2^ at 0.05 mA/cm^2^	--	--	[37]
LIG + PEDOT	115.2 F/g at 0.5 A/g	--	--	[38]
NiO-doped LIG	174 F/g at 5 mV/s	5.6 Wh/kg	429 W/kg	Present

## Data Availability

Not applicable.
